# An open chat with… Josep Rizo

**DOI:** 10.1002/2211-5463.13746

**Published:** 2023-12-14

**Authors:** Ioannis Tsagakis, Josep Rizo

**Affiliations:** ^1^ FEBS Open Bio Editorial Office Cambridge UK; ^2^ Department of Biophysics University of Texas Southwestern Medical Center Dallas TX USA; ^3^ Department of Biochemistry University of Texas Southwestern Medical Center Dallas USA; ^4^ Department of Pharmacology University of Texas Southwestern Medical Center Dallas TX USA

## Abstract

Josep Rizo is a Professor of Biophysics, Biochemistry and Pharmacology at the University of Texas Southwestern Medical Center, where he is Virginia Lazenby O'Hara Chair in Biochemistry. He is particularly interested in the study of the mechanisms of neurotransmitter release and intracellular membrane fusion using structural biology, a variety of biophysical techniques and reconstitution approaches. Jose has been a part of the *FEBS Open Bio* Editorial Board since 2021. In this interview, he shares his insights into developments in the field of neurotransmitter release, describes his move from Spain to the United States, and discusses how sometimes you need to use both logic and scientific hunches.

## Graphical abstract

Josep Rizo is a Professor of Biophysics, Biochemistry and Pharmacology at the University of Texas Southwestern Medical Center, where he is Virginia Lazenby O'Hara Chair in Biochemistry. Here, he shares his insights into developments in the field of neurotransmitter release, describes his move from Spain to the United States, and discusses how sometimes you need to use both logic and scientific hunches.

## How did you first get into biophysics and neuroscience?

Well, it's kind of two different stories. Basically, in Spain college is very focused, so when I started my studies, I wanted to know how the world functions, how everything works. I thought that it was molecules and chemistry, so I studied chemistry in college, specialising in organic chemistry, and they gave me a lot of chemistry with some physics and maths. Then I really liked quantum mechanics and statistical mechanics, so I realised that to really understand how the world functions, I would need to learn theoretical physics. So, my PhD was in organic chemistry and making peptides. But then at the same time, I did my college degree in theoretical physics. So I studied a lot of quantum mechanics, statistical mechanics, quantum field theory, elementary particles, general relativity, all the very core stuff.

And then I realised it was a little bit late for me to become a fully‐fledged physicist. I also like to do experiments – theoretical physics is wonderful, but you need to be part of a 3000‐person team to be able to do one experiment in your life. So, then I decided to do my postdoc in Lila Gierasch's lab where she was using NMR and other biophysical techniques, including studying peptides, and as you know, some peptides are models for protein folding while others are involved in protein export. And so that's how I became a biophysicist—using my chemistry and physics background to understand problems of biological interest, with Lila Gierasch in Southwestern. That was from 1989 to 1994.

I became very enthusiastic about solving the protein folding problem. One of the things I actually learned by myself was to do protein NMR; at the time, there were new techniques that allowed people to solve structures by NMR. And in 1993 I was talking to Lila Gierasch, my mentor, about what to do in the future and I said, ‘I like the protein folding problem but I don't have ideas that are really different from what you are doing,’ and I didn't want to compete with her. And then she said, ‘well, maybe you can find a small protein that one of your crystallographer friends has and maybe you can do something; it was a very general comment.’

I was really lucky that Brian Sutton, who was a graduate student in Steven Sprang's lab at that time, was passing by and he heard the conversation; he said, ‘hey, I have this protein that we believe is a calcium sensor, but it crystallised and when we add calcium, the crystals break.’ And then he said, ‘maybe by NMR I could study it in solution with calcium.’ And at that moment, I did not know what a synapse was, I had no training in neuroscience, no biological training, but I started to read and I could see it was very interesting, how the brain works, how the nerves communicate with each other. Then I met Tom Südhof and we immediately clicked and realised that we could really complement each other. That's how I started to work in neuroscience. That was 1993; my lab was started in 1995 and I've been working on this since then.
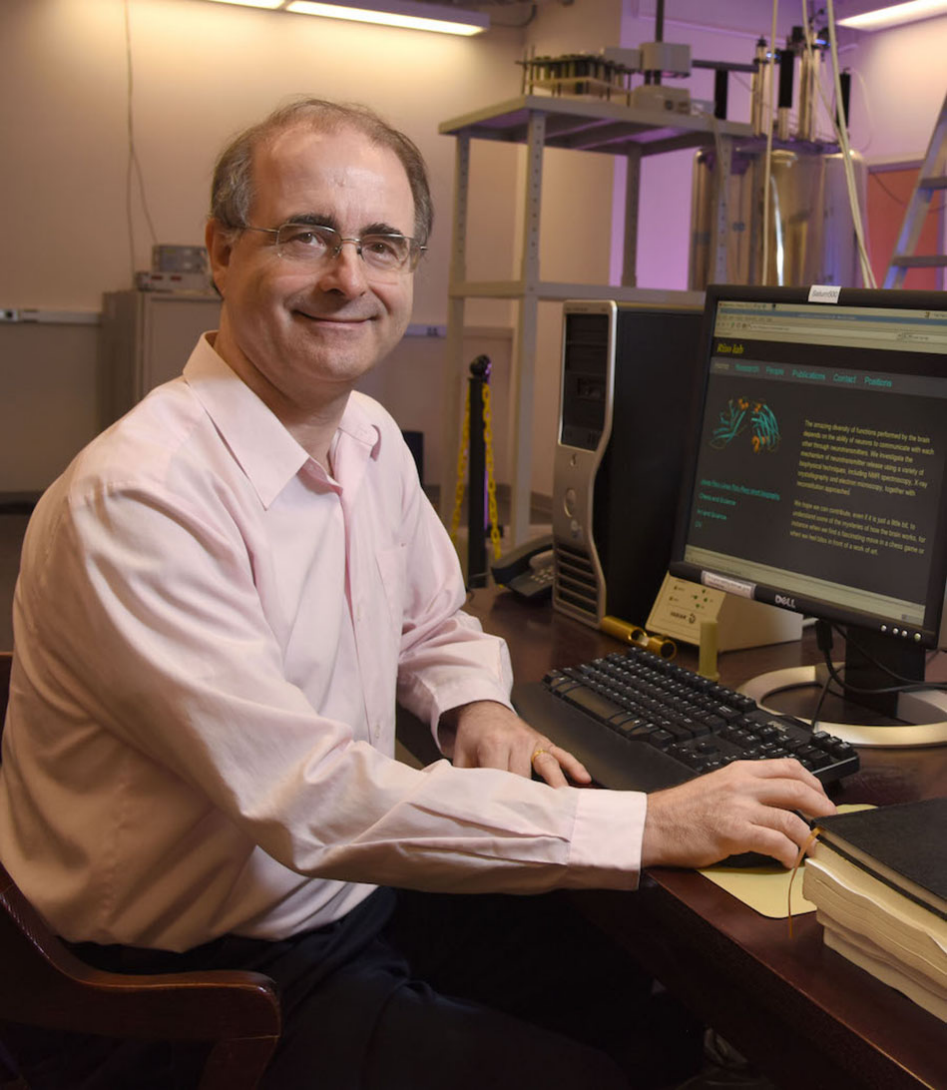


## Did that interest in physics take you from Spain to the USA?

Well, the truth is that I tried to do a postdoc at a good lab in the USA. I received advice from people and applied to two places: one was in the Netherlands with Robert Kaptein who was doing protein NMR, and the other was Lila Gierasch in Dallas. I knew someone who had done a postdoc in New York and he said, ‘you really need to go to work with Lila Gierasch.’ Both of them [Robert Kaptein and Lila Gierasch] said if I can get a fellowship, they would take me in their lab. But then a friend of mine in the US advised me that you really will succeed in the US. I got my fellowship and so I went to work with Lila. At that moment it was basically just the idea of doing a postdoc in America.

## What were the challenges and opportunities in the transition from Spain to the USA?

Well, when you move to the USA it's very different. In particular, Dallas is a completely different city from Barcelona because everything is very spread out, you have to drive everywhere. Also, culturally we are very different from Americans, and so suddenly any European I met seemed so similar to me compared to American people.

Americans helped me a lot, so I cannot complain at all, they really gave me opportunities and they made me feel very welcome. The people in the lab were very helpful; they really helped me to get settled. So, I really think that America was very welcoming. If there is one great thing in America it's that they really welcome foreigners, even though there is all this hullabaloo about immigration and so on. But I think every country tries to prevent immigration except maybe all the countries where they need people.

## Do you feel that having this inter‐disciplinary background (biophysics and neuroscience) has given you unique insights into approaching research questions compared to other researchers?

Yes. That's part of the reason I wanted to work on protein folding because I could see that there were fantastic people working on protein folding, but they didn't know statistical mechanics. And they were talking about pathways and Levinthal's Paradox and things like that, and I could see that most people didn't really understand.

I think in terms of what I've been doing with neurotransmitter release, I feel that I've been really blessed by having a strong physics and chemistry background, but also a strong biophysics background with Lila Gierasch. Working on protein folding has been really important for me to understand how the molecules involved in neurotransmitter release move and change to drive the process. So, there are a lot of things; it's not just the structural biology, it's not only taking pictures. We need to understand how things can change, how interactions may be strong but they have to break, and the effects of mutations. So, you need to have this dynamic view and I think that my training with Lila Gierasch was really fundamental for me to be able to make a big impact in our field.

## Is there any progress towards developing wet lab techniques that are missing, or is it still a bottleneck?

I don't think we are even close to being able to observe the process of membrane fusion, a bunch of lipids moving, in real time at atomic resolution. Cryo‐electron microscopy is a wonderful technique, but in order to really be able to have a high resolution you need to take hundreds of thousands of pictures of the exact same particle or a very similar particle, and then you average to get the high‐resolution structure.

So, if you're observing a fusion reaction, it will be different every time it happens; with electron microscopy, you can observe what's happening at low resolution, but you really cannot see it at an atomic resolution – apart from individual frames. It's impossible to get an average of hundreds of thousands of frames because you don't have hundreds of thousands of identical frames. So, right now there is no technique for this at all.

There are many probes, there are many ways to look at different aspects of fusion, and of course my lab and others are using them, but there is no way to examine it at atomic resolution in a dynamic fashion. The good thing is that with the molecular dynamic simulations, I see that things are really making a lot of sense. I think the force fields are very good and you can correlate the results of the simulations with what you observe with experiments that have been performed, and it makes a lot of sense. I did get full fusion and I'm working on a paper about this.

## How does computational biology help address questions not possible using wet lab techniques? And how has computational biology aided your understanding of neurotransmitter release?

So, you have AlphaFold as a computational biology method. In our case, AlphaFold is not helping much because we already have the structures. If it was ten years ago, AlphaFold could have helped a lot. In everything we are studying now, we mostly need to understand weak interactions and AlphaFold doesn't do well with that for the moment, but at some point, down the road it may help, but right now it doesn't. So, I think molecular dynamics in that sense, yes, it's a computational technique, but it's more about trying to reproduce what happens in reality using these physical forces. And there are lots of approximations. I was actually very sceptical when I started to do this; I was thinking that it might give me some insights and it would help me to visualise things, but I was not expecting that it would be as informative as it has been.

## What do you think are some new directions that the field is heading or maybe could expand to?

Well, I think that the big direction is clearly the regulation of neurotransmitter release. So, the big thing that we have already started to work on, and Jim Rothman is also working on, is Munc13, which is essential for release but it's also a master regulator. Release happens in something called the active zone that is formed by Munc13 and several other homologous proteins. These control the release probability and controlling the release probability is one way to process information in the brain. So, clearly the direction for the future is regulation.

## Researchers often use logic–but would you say you sometimes have to use intuition to make future decisions?

Yes, definitely, especially when things don't make sense to you. You try your logic and then sometimes some results do not make sense: they don't fit the logic. And that's when people say, ‘think outside the box.’ I don't like that term because people use it way too often, and there are too many people that always tend to think outside the box, and it's really bad—they're too far outside the box! But then sometimes when you're stuck, I think the idea is that you should use logic first, but then it often happens that the logic doesn't work and then you need to think of what may be wrong. You have your framework that is trying to explain things but it's not, so it's probable that something is missing or something is wrong, and then you need to try to go with something else and intuition is definitely very important at those times.

## Is there such a thing as a scientific hunch?

Well, sometimes logic is not going to help you. You have a model in mind, you keep testing it and some things fit, other things don't fit, but you're not sure if you should abandon the whole model. And you need to have enough things that don't fit to say, ‘no, we need to really change this.’

So, I think it's a combination of logic and hunch. Sometimes you make the big jumps with a hunch, but it doesn't come out of the blue, it doesn't come completely without logic. It's after trying a lot with logic that then sometimes you have to make that big leap.

## How big a role would you imagine having a good short‐term memory has in achieving success in a PhD or maybe in a scientific career overall?

I think that long‐term memory is more important for research. Of course, short‐term memory is important for everything you do every day; imagine if you read a paper but then forgot it the next day! And if you don't have short‐term memory you will probably not create long‐term memories anyway. But long‐term memory is also important because you need to be able to remember things that happened ten years ago: many times, there are certain things that you just can't explain and you leave them sitting in some corner of your mind. Then suddenly one day, the answer comes to you after ten years!

## Do you think winning awards is instrumental for the progression of a scientific career?

Absolutely not. I think that awards are nice. But the problem with awards is that you need to convince people who don't understand your science that well that the work was good, which means that not only do you have to do good science, you also have to carry out some marketing. Normally the people who receive awards have done something interesting. But you can also see very uneven CVs: some people may have 20 papers and 20 awards, and someone else has 200 papers and two awards. And now you have to think: who is the better scientist? It's case by case, but I would always bet more on the person who has lots of papers and fewer awards than the person who has a lot of awards and not so many papers. But of course, there are always exceptions–maybe those 20 papers are fantastic.

Anyway, I think it's okay to have awards, but I don't worry too much about it. When I introduce speakers, I barely talk about awards; normally, I always talk about the science, which I think is what's important. And if that brings you awards, that's wonderful.

## Why did you decide to join the *FEBS Open Bio* Editorial Board?

I'm on a couple of other editorial boards, but normally they don't ask me to participate too much. With *FEBS Open Bio* it was because the Editor‐in‐Chief asked me if I wanted to be on the board and I've known him for a long time. And I like to support journals that are run by active scientists. I think that these journals that are not run by active scientists will eventually disappear because most of them are not very well run, although of course there are always exceptions. I've had some very good experiences with some of the Nature journals, but also some very bad ones.

I think in general, the moral should be that active scientists should be making the decisions and not people who are out of touch with research. They are still scientists, but they get out of touch and I think that people who are very worried with impact factors really lose sight of what is really good science or not. People who are still active scientists are much more likely to be in touch with the reality of science.

## Would you consider making your research findings available as a preprint—and would you review the preprints of other people?

I've sometimes put papers on bioRxiv before publication in a journal. Of course, going into bioRxiv and adding reviews to an unpublished paper takes a lot of time, because if you want to do it well you should read the paper carefully. I do that as a reviewer for journals. I think that I might review pre‐prints some day if I retire, but for now I think it's more important that I spend time carrying out peer review for journals.

## Why is it more important if it's done for a journal than for a preprint paper?

I think that ideally peer review should be coordinated. I don't like elitism, I don't like impact factors. So, normally when I review papers, I don't care what journal sends it to me. And if they ask me if it's important for the journal, I normally don't answer that question. I give my impression of how important the research is. Basically, I think papers should be peer reviewed, and I don't think putting something out there on bioRxiv and hoping that people will review it is a good system. I think the better system is to send it to a peer‐reviewed journal that hopefully will do a good job and ensure the work is critiqued.

## Have you heard of Plan S, an initiative for open‐access publishing? Do you think this would be a good initiative to be accepted by all journals or do you think there could be some pitfalls with this initiative?

So, the idea is that any paper that is published should be open access immediately? I think that's great. I understand that [for‐profit] journals want to make money, but then you can just charge a publication fee like the open‐access journals are doing, as long as it's moderate, and not like some of these journals that are charging $10 000 to publish a paper. This is so unethical. We teach students about ethics, but never talk about some of the biggest problems we have in science. I think that journals that charge $10 000 for open access publication should be out of the picture. They're anti‐science and it's the top journals, the very top journals that are doing this – so they should disappear, ideally.

## Can you give me an example of a thrilling experience that triggered a similar feeling outside the lab?

I love reading non‐scientific literature, and also scientific literature, and I also spend a lot of time reading novels, history, philosophy and all kinds of things. And I play chess. I usually don't have time to play a long chess game so I play fast three‐minute games at chess.com.

